# Patient safety risks associated with telecare: a systematic review and narrative synthesis of the literature

**DOI:** 10.1186/s12913-014-0588-z

**Published:** 2014-11-25

**Authors:** Veslemøy Guise, Janet Anderson, Siri Wiig

**Affiliations:** Department of Health Studies, University of Stavanger, Kjell Arholms gate, 4036 Stavanger, Norway; Florence Nightingale School of Nursing and Midwifery, Kings College London, London, UK

**Keywords:** Telecare, Homecare, Patient safety, Human factors, Systematic review, Narrative synthesis

## Abstract

**Background:**

Patient safety risk in the homecare context and patient safety risk related to telecare are both emerging research areas. Patient safety issues associated with the use of telecare in homecare services are therefore not clearly understood. It is unclear what the patient safety risks are, how patient safety issues have been investigated, and what research is still needed to provide a comprehensive picture of risks, challenges and potential harm to patients due to the implementation and use of telecare services in the home. Furthermore, it is unclear how training for telecare users has addressed patient safety issues. A systematic review of the literature was conducted to identify patient safety risks associated with telecare use in homecare services and to investigate whether and how these patient safety risks have been addressed in telecare training.

**Methods:**

Six electronic databases were searched in addition to hand searches of key items, reference tracking and citation tracking. Strict inclusion and exclusion criteria were set. All included items were assessed according to set quality criteria and subjected to a narrative synthesis to organise and synthesize the findings. A human factors systems framework of patient safety was used to frame and analyse the results.

**Results:**

22 items were included in the review. 11 types of patient safety risks associated with telecare use in homecare services emerged. These are in the main related to the nature of homecare tasks and practices, and person-centred characteristics and capabilities, and to a lesser extent, problems with the technology and devices, organisational issues, and environmental factors. Training initiatives related to safe telecare use are not described in the literature.

**Conclusions:**

There is a need to better identify and describe patient safety risks related to telecare services to improve understandings of how to avoid and minimize potential harm to patients. This process can be aided by reframing known telecare implementation challenges and user experiences of telecare with the help of a human factors systems approach to patient safety.

## Background

The home is becoming an increasingly important setting for the delivery of healthcare services. Telecare, technology that enables healthcare professionals to remotely care for and support home dwelling individuals, has been suggested as a means of improving home healthcare services [1] and promises to be an important solution to the many challenges facing future healthcare services. The safe use of telecare services is however contingent on complex, dynamic processes [2]. While the implementation of information and communication technologies (ICTs) in healthcare settings has the potential to improve the safety and quality of services [3], it may also introduce potential safety risks [4–6]. Concerns regarding the safety and quality of telecare and other so-called health ICTs can seriously undermine their integration into traditional healthcare services. It is not uncommon, for example, for healthcare professionals to report reservations about providing care at a distance due to concerns about unsafe care processes and poor outcomes for service users [7,8]. Sustained implementation and adoption of telecare tools and services is thus contingent on evidence of its quality, safety and relative advantage to users [6,9]. It has therefore been suggested that attention to patient safety should be an important driver in ensuring integrity in the design, implementation and operation of telecare services [10].

Patient safety incidents can involve actual or potential harm to patients and involve both organisational and individual factors. Poor patient safety is a complex issue with many antecedents but it is widely accepted that adverse events result from systemic features of care across multiple levels, such as those to do with the professionals/team involved, the tasks concerned, the technology and tools used, the work environment, and the organisational setting [11,12]. Threats to patient safety are thus largely understood to stem from the context and conditions of healthcare work, which sees humans acting within complex sociotechnical systems [11]. Accordingly, the goal of patient safety practices is to reduce risk of harm to patients stemming from the structures and processes of care [13].

### Patient safety risk related to telecare services

Information on the safety and quality of telecare systems is inconclusive [2,6,14]. There is indication that patient safety risks exist at a variety of care levels [15], but the extent and consequences of those risks are not fully understood [16,17]. It has been suggested that knowledge is lacking because risks, problems and failures to do with the safety and quality of health ICTs are frequently not reported as such. Rather, emerging risks are explained as unintended or indirect results to do with flawed study design [15] and the potential patient safety consequences of these risks are therefore often not elaborated upon [18]. Furthermore, patient safety is likely being compromised by gaps in current initiatives related to the safe use of health ICTs, such as regulatory requirements and mandatory reporting systems. While many ICT tools for diagnosis and treatment are subject to regulation as medical devices, where reporting of adverse incidents is mandated [19], enforcing such regulations in the homecare setting is challenging [20,21] and often dependent on the voluntary actions of home healthcare providers and patients [19]. Increased transparency and standardisation in the reporting of patient safety issues related to health ICTs is urgently needed to improve the evidence base [2,15,19,21].

### Patient safety research in the homecare setting

In the homecare setting, as in healthcare settings elsewhere, adverse events are thought to result from an alignment of several factors that alone may not be sufficient to result in harm [22]. It is however noted that due to the largely unregulated and uncontrolled nature of the home as a site for healthcare processes, patient safety risks found in the homecare setting are often different from those seen in institutional care settings [23]. For example, patient safety in homecare is inextricably linked to relationships and interactions between patients, informal caregivers and formal healthcare providers [23,24]. It is possible for homecare staff or informal caregivers to contribute to adverse care events [1]. Moreover, the capability of the patient (and informal caregiver) to manage their own healthcare needs and participate in their own care is an important aspect of patient safety in the homecare setting [23].

More work is needed to understand the causes and circumstances of adverse events in the homecare setting [23,24]. Existing models and frameworks may be unsatisfactory for use in this setting and may cause safety problems in the homecare sector to be overlooked [24,25]. Research is therefore needed that reflects the multidimensional aspect of patient safety, where consideration is given to the unique conditions of the home as a site for the provision of healthcare, as well as to the roles of patients, caregivers and providers as key players in the larger system [22]. Human factors and ergonomics approaches have been suggested as a suitable means for conceptualising and examining safety and quality concerns in home-based healthcare, as it implies consideration of interdependencies and interactions between humans and a broad range of relevant socio-technical factors [22,26,27]. Within a human factors framework, the home can be conceptualised as a complex, holistic work system where the different yet interrelated elements of the system come together to influence work-flow and care processes over time, which again influence a range of patient, provider and organisational outcomes [12,27,28]. The use of such a framework may therefore contribute to improved system performance and ultimately support the overall quality and safety of telecare services in the home [29].

### The role of training in the mitigation of patient safety risks

Sound competence in the use of telecare services is a fundamental requirement for the provision of ethical and safe healthcare [30]. Education and training for users is widely acknowledged as an important mitigating factor in reducing patient safety risks associated with telecare use [6,31–33]. For example, training for telecare providers facilitates standardisation of working practices, which helps ensure safe and proper use of services [10,34]. Telecare training should furthermore include considerations of professional accountability, risk assessment and risk management related to its use [35,36]. A focus on patient safety in staff training can also help create an organisation-wide culture of safety [31]. Although there is a clearly recognised need for specialised skills and knowledge in the provision of telecare services [37–40], however, research suggests a pervasive lack of educational programs and formal curriculums aimed at telecare practitioners [41]. According to one study, the vast majority of telecare providers are learning on-the-job rather than from formal training sessions and are thus not formally certified for telepractice [42]. Despite longstanding calls for a minimum standard of required competencies and training for telecare practitioners [37,43], and for the inclusion of ICT related skills and knowledge across healthcare curricula [44], it appears that a lack of informatics content remains in educational programs for healthcare providers [45].

### Aim

A systematic review of the literature was undertaken to identify patient safety risks associated with the use of telecare services in the homecare setting and to investigate whether and how these patient safety risks have been addressed in training. This review is part of a research project aimed at developing and evaluating telecare training programs for healthcare professionals and elderly service users in the home healthcare setting [46]. Its purpose is to inform the development of these training programs. The review questions were:

1. What are the patient safety risks associated with telecare use in homecare services?
2. Have these patient safety risks been addressed in training for healthcare staff and, if so, how?

The SEIPS model of work systems and patient safety [12], a human factors systems approach to the examination of patient safety concerns in complex healthcare settings, was used to frame and analyse the review findings.

## Methods

### Design

A systematic search of the literature was undertaken to identify patient safety risks related to telecare use in the home, and to investigate how these patient safety risks have been addressed in telecare training. Inclusion criteria were as follows. As the focus of the larger study of which this review is a part is on telecare for older people, the study population and setting of interest was restricted to adults (ages 18+) living at home receiving homecare. The type of care model or service of interest was telecare. The terminology related to the use of ICTs in health and social care is inconsistent [47,48], so a number of terms related to ‘telecare’ were used in the search, including ‘telemedicine’ and ‘telehealth’. Of specific interest to our review were studies on the use of videophone or video conferencing equipment [46] and these and associated terms were also used in the search.

While ‘telecare’ and related terms are often taken to implicitly refer to care delivered in the homecare setting [48], an initial scoping review revealed that it was necessary to include search terms related to healthcare delivery in the home to narrow down the search results to the actual setting of interest. Also, ‘simulation’ was included as a search term together with ‘training’ and education’ since simulation is widely acknowledged as a particularly valuable approach for promoting an overall culture of safety and teaching the knowledge and skills necessary for safe clinical practice [32,49–51]. The scoping review furthermore revealed an absence of RCTs or cohort studies in this field, therefore a range of primary research studies and reviews featuring a variety of qualitative and quantitative study designs were included for review. Studies were excluded if not concerned with adults, not concerned with telecare, or not from the homecare setting, as were studies describing participants’ feelings of being safe and secure in relation to telecare use. Also excluded were editorials and other opinion pieces. The relative risks and benefits of telecare were not investigated in this study as the focus was on identifying evidence about patient safety risks and how this is addressed in training.

### Search methods

Six electronic databases were searched: Medline, CINAHL, ISI Web of Knowledge, Academic Search Premier (ASP), Scopus and Science Direct. Searches were performed in November and December 2013. Search results were restricted to those published in English only, but no limits were set on publication dates. See Table 1 for search terms and structure of the search.

**Table 1:** **Search terms and structure of search**

#1	Telecare OR telehealth OR telemedicine OR telehomecare OR telenursing OR videophone OR video conferencing OR video visits OR virtual visits OR televisits OR telecommunication
#2	Patient risk OR patient safety OR patient harm OR quality OR adverse event OR undesired event OR medical error
#3	Homecare OR Home care services OR home-based care OR community health service OR community dwelling
#4	Training* OR education* OR simulation*
#5	1 AND #2 AND #3 AND #4
#6	Limit #5 to English language

* = wildcard filter applied.

In addition to the electronic searches, non-protocol driven searches [52] were undertaken in January 2014, incorporating reference and citation tracking as well as hand searches of key items and other resources known to the authors. Reference management software was used to organise and store search results. All three authors participated in the process of selecting eligible items for inclusion. Author VG performed the searches and undertook the initial screening of titles and abstracts against inclusion criteria, with authors JA and SW independently participating in the second screening of titles and abstracts. VG then undertook the read-through of selected full-text articles. Where there was question of inclusion eligibility, JA and SW were consulted independently to assess full-text item suitability.

### Search outcome

**Figure 1:**
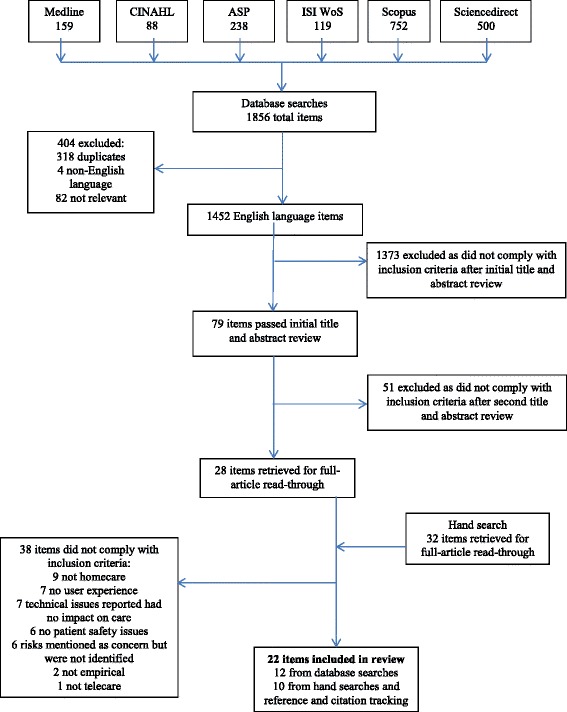
**Flowchart of article selection process.**

The database searches identified 1856 items, while the hand search identified 32 titles, a total of 1888 items. 404 database items were excluded prior to the initial title and abstract review, as they did not fit inclusion criteria. A further 1373 items were excluded after the first title and abstract review. The remaining 79 database titles were subjected to a second title and abstract review by all three reviewers, leading to the exclusion of another 51 items. The remaining 28 items from the database search and 32 items from the hand search (60 items in total) were retrieved for a full article read-through, resulting in a total of 38 further exclusions. Reasons for exclusions of full-text items included items not being concerned with the homecare setting (or it is unclear which findings apply to this setting), not being related to patient safety issues, or not being concerned with telecare or having no actual experience of telecare use among participants (or having no reported user experience). Items describing technological issues that had no (reported) impact on clinical care or patient safety were also excluded, as were items that mentioned the safety of telecare use as a concern without identifying the nature of safety risks. Twenty-two articles were included in the review. See Figure 1 for details on the article selection process.

### Data extraction

The data extraction tool was designed to best enable answering of the research questions and to facilitate quality appraisal. The data extraction process accordingly assessed study design, purpose and aims, and methodological rigor and validity. Other information extracted was related to participant numbers and characteristics; type of telecare service/system described and purpose of the technology; risks to patient safety associated with telecare use; training and education initiatives that address patient safety risks associated with telecare use; and content, form and other recommendations that can inform the design of telecare training programs for healthcare professionals. Author VG extracted the data verbatim, before it was discussed by all authors and synthesized into themes according to the review question.

### Quality appraisal

The quality appraisal process was completed in a similar way to that described by Brewster et al. [53]. Quality assessment was done using different tools according to the type of study design and methods used. The qualitative studies were assessed using the Critical Appraisal Skills Programme (CASP) quality assessment tool for qualitative studies [54], reviews were assessed using the CASP quality assessment tool for reviews [54], whereas quantitative studies were assessed using the quality assessment tool for quantitative studies developed by the National Collaborating Centre for Methods and Tools (NCCMT) [55]. Mixed-method studies were appraised partly using the qualitative CASP tool and partly with the quantitative NCCMT tool. As part of the data extraction process, study quality was appraised by assessing the suitability of study purpose to study design and methods, as well as an appraisal of methodological soundness. No studies were excluded based on quality criteria, though the appraisal did find some inadequate descriptions of the methods and processes used. Overall quality of papers was found to be acceptable, with appropriate methods used to address clearly stated research goals.

### Synthesis

Narrative synthesis was undertaken to organise and synthesize the findings. Due to the range of research designs used in the studies included for review, an approach suitable for use with both qualitative and quantitative data was needed to synthesize the extracted data. Narrative synthesis has been recommended for reviews encompassing findings from multiple, heterogeneous studies when statistical meta-analysis or meta-ethnography alone are not viable options [56]. Narrative synthesis is characterised by a textual approach to the process of synthesis, relying on the use of words to summarise and explain findings. The approach involves a preliminary synthesis by way of an inductive thematic analysis of the individual study results. Thematic analysis comprises identifying, listing, tabulating and counting themes according to the review question(s), to enable description of patterns across included studies and, importantly, exploration of relationships within and between studies [56].

The initial synthesis by thematic analysis was conducted by VG, with further analytical input from SW and JA. This process entailed free coding of findings from the individual studies; construction of descriptive themes based on these codes; and a final synthesis of descriptive themes with reference to the five analytical categories of the SEIPS model for work systems and patient safety [12]. This framework consists of the following interrelated factors: the individuals involved (including patient and provider characteristics and capabilities); the tasks involved (such as the nature of care tasks); the tools and technology in use (including the usability of technology); organisational factors (including culture, structure, rules and procedures); and the environment within which the work is carried out (including features of the physical, social and external environments) [12,28]. In addition to categorising emerging patterns across studies in this way, relationships between identified patient safety risks and factors such as study design and purpose, methods used, study participants, and the telecare systems/interventions described were explored [56].

All data included in this review were previously published and publicly available. The study therefore did not require ethical approval.

## Results

### Included studies

Here we describe the included studies, before detailing identified patient safety risks and addressing the training aspect. Twenty-two articles published between 2001 and 2014 were included for review. Only one study had the investigation of safety issues associated with a home-based telecare service as an expressed aim. This study was concerned with the safety, security and privacy of a telecare monitoring system [57]. Two other studies had a focus on risk management and human factors issues related to the design and use of telecare. One of these was a multiple-case study investigating how project risk management was applied and shaped outcomes in a range of projects using mobile technology software to plan and organise homecare nursing activities [58]. The other was an analysis of human factors issues necessary for the design and implementation of safe and effective home-based consumer health IT applications [59].

The 19 remaining studies were variously concerned with the evaluation of acceptability, effectiveness, reliability and impact of home telecare systems, including exploration of user experiences and perspectives, as well as investigation of factors associated with implementation and use of telecare systems. The majority of included studies (11) had a qualitative design featuring observation, interviews and/or focus groups as data collection methods. Three were case studies featuring qualitative or mixed-methods, three were mixed-method studies, three had a quantitative design, and two were systematic reviews. Four studies had patients as participants, 8 had staff as participants, and 8 had a mix of patient and staff participants. The majority of these studies were concerned with the views and experiences of patients and/or staff. The majority of telecare interventions described were systems or devices for the purposes of vital signs monitoring (11), 8 featured both telemonitoring devices and systems for communication, two were systems for communication only, whereas one was an application to aid care planning. In the main, the telemonitoring systems were concerned with clinical monitoring and management of blood pressure, blood glucose and chronic obstructive pulmonary disease (COPD) symptoms, though 14 studies did not specify the healthcare problems concerned. Between one and six risks to patient safety were identified in each of the included studies.

### Risks to patient safety

Findings have been structured into the following 11 categories, presented in descending order according to how many times they were identified in the included articles: Change in the nature of clinical work (15); Lack of patient and/or staff knowledge and understanding (13); Technology issues (9); Changes to staff workload (8); Accessibility issues (3); Lack of guidelines (3); Patient dependency (3); Patient anxiety (2); Poor system integration (2); Poor patient compliance (2); and nature of homecare environment (1). Each category is explained in more detail below and in Table 2.

**Table 2:** **Overview of included articles**

**Author and year**	**Purpose of study**	**Study design (& methods)**	**Study participants**	**Type of telecare service/system described**	**Source of potential risk to patient safety**
**Brewer et al. (2010) [** 57 **]**	To investigate perceptions of the safety, security and privacy of a telecare monitoring system	Survey	127 different stakeholders	Telecare monitoring for adults with developmental disabilities	Change in nature of clinical work
**Brewster et al. (2014) [** 53 **]**	To analyse the impact of telehealth implementation on front-line nursing staff	Systematic review	Nursing staff	Telehealth technologies for the management of COPD and CHF	Change in nature of clinical work
					Changes to staff workload
**de Lusignan et al. (2001) [** 72 **]**	To examine the acceptability, effectiveness and reliability of home telemonitoring	Controlled pilot study	20 patients	Pulse and blood pressure devices, video consultation equipment	Technology issues
					Patient dependency
**Essén & Conrick (2008) [** 71 **]**	To explore constituents and challenges related to innovation of technology-based services in the long-term homecare sector	Case study (Focus groups, interviews, observation)	Home-help managers and home-help staff and 10 operational/managerial staff	Sensor-based telemonitoring system	Lack of user knowledge (patients and staff)
					Changes to workload
					Lack of guidelines
**Hanley et al. (2013) [** 74 **]**	To explore experiences of users taking part in a RCT of remote blood pressure (BP) tele-monitoring. To identify facilitators or barriers to the effectiveness and routine uptake of the intervention	Qualitative interview study	25 patients, 11 nurses and 9 doctors	A home BP monitor and mobile phone technology for transfer of BP readings via SMS to a secure website	Patient anxiety
					Patient dependency
					Poor system integration
					Changes to workload
					Accessibility issues
**Hibbert et al. (2004) [** 65 **]**	To document responses of nurses using telehealth equipment and identify service integration issues	Ethnography (observation)	12 nurses	A home telehealth nursing service for COPD patients, using videophone and vital signs monitoring	Technology issues
					Change in nature of clinical work
**Hopp et al. (2006) [** 68 **]**	To examine staff perceptions of opportunities and barriers of home-based telemedicine services for chronic illness care	Qualitative interview study	37 direct telemedicine providers, primary care providers and hospital administrators	Store-and-forward devices, video conferencing devices	Lack of user knowledge (patients and staff)
					Technology issues
					Poor patient compliance
					Change in nature of clinical work
					Changes to workload
**Horton (2008) [** 69 **]**	To evaluate a home telecare service for COPD patients	Qualitative study (focus groups and case study)	4 home care team and social care staff and 6 patients	Daily monitoring of patients’ condition via call centre with community response service	Technology issues
					Lack of user knowledge (patients and staff)
**Lu et al. (2014) [** 70 **]**	To describe the use of home telehealth care for chronic disease management from users’ perspective	Qualitative study (focus groups and interviews)	20 patients	Telemonitoring of BP and/or blood sugar, provision of health care/consultations with healthcare professionals via computer or telephone	Lack of user knowledge (patients)
**Mair et al. (2008) [** 60 **]**	To perform a process evaluation of a RCT of home telecare for the management of COPD	Qualitative interview study	9 patients and 11 nurses	A videophone link and attachments for remote physiological monitoring of vital signs	Change in nature of clinical work
					Changes to workload
**Marziali et al. (2005) [** 77 **]**	To assess frequencies of reporting adherence to professional practice standards and research ethics in studies of technology-based home healthcare programmes	Systematic review	107 articles describing studies on the use of telecare, featuring a variety of staff and/or service users	Medical symptom monitoring using synchronous technology	Lack of guidelines
**Nilsson et al. (2010) [** 73 **]**	To describe two district nurses’ experiences of using ICT to communicate with chronically ill people in their homes	Qualitative interview study	2 district nurses	An electronic messaging system to communicate with patients	Technology issues
**Radhakrishnan et al. (2012) [** 61 **]**	To explore perceptions on effectiveness of telehealth for heart failure management in a homecare setting	Mixed-methods (focus groups, interviews and questionnaire)	44 nurses and 4 patients	A centralized model of daily telemonitoring of vital signs by a telehealth nurse, with in-person follow-up if needed	Patient anxiety
					Patient dependency
					Lack of user knowledge (patients)
					Changes to workload
					Change in nature of clinical work
					Lack of guidelines
**Roberts et al. (2012) [** 75 **]**	To evaluate a telehealth programme for long-term conditions	Mixed-methods (questionnaire and interview)	Patients, carers and 10 medical, healthcare and managerial staff	Home-based touch screen facilities for clinical monitoring for COPD and hypertension patients	Changes to workload
**Sandberg et al. (2009) [** 62 **]**	To understand the experiences of providers and the factors perceived to contribute to the success of telehealth interventions and user satisfaction	Qualitative interview study	10 telemedicine providers (nurses and dietitians)	A telemedicine unit with video-conferencing, blood glucose and blood pressure readings and educational materials	Technology issues
					Lack of user knowledge (patients)
					Change in nature of clinical work
**Shea & Chamoff (2012) [** 67 **]**	To examine the relationship between communication and information integration into the daily lives of patients with chronic illnesses and offer best practice recommendations for telehomecare nurses	Descriptive, correlational study	43 patients and 9 telehomecare nurses	Telemonitoring; patients interact with nurses using a telestation that collects and transfers data via telephone lines	Lack of user knowledge (patients and staff)
**Sicotte & Paré (2011) [** 58 **]**	To investigate how project risk management was applied in 9 mobile computing projects and how it shaped project outcomes	Case studies (mixed-methods)	57 project leaders, nurse users and nurse pilots from 9 homecare units	Mobile technology software for planning and organization of homecare nursing activities	Technology issues
					Poor system integration
					Changes to workload
**Skär & Söderberg (2011) [** 63 **]**	To describe influences, benefits, and limitations in using ICT to meet chronically ill patients’ needs when living at home	A descriptive, exploratory pilot study	2 patients, 1 relative, 1 district nurse and 5 personal assistants	An application for information and communication between chronically ill people and the district nurse	Change in nature of clinical work
					Technical issues
**Wälivaara et al. (2011) [** 64 **]**	To describe the reasoning among general practitioners about the use of mobile distance-spanning technology (MDST) in care at home and in nursing homes	Qualitative interview study	17 doctors	Mobile distance-spanning technology for communication and diagnostic purposes	Change in nature of clinical work
					Lack of user knowledge (patients)
**Wälivaara et al. (2009) [** 66 **]**	To describe how people in need of health care at home view technology	Qualitative interview study	9 patients	Distance-spanning technology with mobile devices to measure vital signs	Poor patient compliance
					Lack of user knowledge (patients)
					Accessibility issues
					Change in nature of clinical work
**Young et al. (2011) [** 76 **]**	To seek accurate patient perspectives about benefits and challenges of a care coordination/home telehealth program	Mixed-methods (survey and interviews)	25 patients	Messaging devices, monitoring and measuring devices, video-phones and PCs	Accessibility issues
**Zayas-Cabán & Dixon (2010) [** 59 **]**	To analyse human factors and ergonomics issues encountered during the design and implementation of home-based consumer IT applications	Case studies (analysis of documents and discussion notes)	5 home-based consumer IT application projects	Various IT applications including videophone, messaging systems and health monitoring devices	Technology issues
					Unsafe device arrangements

*Change in the nature of clinical work* refers to patient safety risks associated with the tasks healthcare staff traditionally perform in the homecare setting and was the safety issue that featured in most studies. Such risks are a result of the lack of in-person care and hindrances presented by the use of ICT instead of face-to-face care. Examples include studies where the lack of in-person care was found to hinder thorough clinical assessment [60–63] and good treatment decisions [53,64] on the part of healthcare professionals, or where the lack of in-person care was considered inappropriately risky in case of an emergency [57,65,66], particularly with perceived acute patients [60]. The use of ICT was also seen to have a negative impact on the traditional clinical relationship. The use of technology adversely affected staff-patient interaction [53,60] and hindered good communication [67] and the process of ‘getting to know’ the patient [63,65], making it harder to develop good clinical relationships [68]. As a consequence, healthcare professionals regarded the use of telecare to be less safe than standard care [53], with some preferring in-person care for safety reasons [68].

*Lack of patient and/or staff knowledge and understanding* of system functionality and performance was another major patient safety risk identified in the literature. Many studies described a lack of knowledge, skills and/or understanding on the part of patients [66–70], which can compromise their safety [71] in various ways. Lack of user knowledge can for example lead to an inability to use the telecare system properly [62] or overconfidence in the capabilities of the system [64]. A consequence of this is that patients do not report their symptoms to staff, thinking that the system will relay the measurement information directly [61]. Studies also reported lack of staff knowledge of how to interpret and respond to data [71], due to an underestimation of the knowledge needed to use the technology [68] and lack of staff training [69]. There was also an example of a lack of shared understanding of the goals and purposes of a telecare system, where staff and patients interpreted system functionalities differently, leading to communicative misunderstandings [67].

Patient safety risks to do with *technology issues* was a feature of nine studies. Poor technical quality of systems was for example found to hinder good and timely communication between staff and patients [63,65,72,73]. While user interface issues were not specifically mentioned in any of the review studies, other issues with poor usability of technology such as reduced ease of use and low user-friendliness affected several studies [58,68,69], whereas poor reliability (e.g. undependable examinations and measurements) interrupted continuity of treatment in one study [62] and led to patients avoiding the use of the technology in another [59]. Another significant issue that emerged in several studies was direct *changes to staff workload* and associated changes to staff roles and responsibilities [53]. Healthcare staff were concerned that the often unforeseen, added workloads brought about by the use of new telecare systems had a detrimental effect on their ability to perform traditional tasks and responsibilities [58,60,61,68,71,74,75].

The following patient safety risks were noted in between one and three studies. *Accessibility issues*, seen in three studies, refers to problems or delays when trying to contact staff or patients through telecare technology [74,76], as well as problems in receiving user support [66]. A *lack of guidelines*, hereunder user protocols, clinical practice guidelines and quality assurance systems for the delivery of telecare services, was seen in three studies, one of which was a systematic review which noted this to be a broad ranging issue across the homecare sector [61,71,77]. Patients becoming *dependent* on the technology, potentially putting them in a ‘sick role’ and impairing their ability to self-manage their condition, was a problem also seen in three studies [61,72,74], whereas two studies noted that the in-home monitoring of signs and symptoms provoked *anxiety* in some patients and so the service was discontinued [61,74].

Two studies noted *poor system integration*, where the new telecare system was not integrated with existing systems [58] such as the electronic patient record system, thus hindering multidisciplinary working and communication between healthcare staff [74]. *Poor patient compliance* was also noted in two studies, which found that patients who were not motivated to participate in their own care via telecare [68] disassociated from the technology and abdicated responsibility for its use to healthcare staff [66]. Finally, *environmental factors* contributed to patient safety risks in one case study, where telecare devices were not properly set up for ease of use. Unsafe device arrangements led to exposed cords representing a tripping hazard [59].

### Addressing patient safety risks in telecare training

None of the studies found describe training initiatives or whether patient safety risks are addressed as part of telecare training. Twelve of the 22 included articles do however mention the importance of training or education initiatives for sound use of telecare services. Six studies conclude that more training and education is needed to adequately prepare telecare users to take part in their own care and promote greater understanding and acceptance of telecare [58,62,67,68,70,76]. A further two studies note that training must be a part of telecare implementation [53,57]. Based on their study findings, five articles suggest ideas about what issues training should cover and offer observations on the form training should take. In addition to instructions on how to use the technology and how to resolve technical problems [70], telecare training should have a broad educational focus on underlying systems and services [68]. Training should cover new ways of interacting with patients and colleagues and should address changes to staff roles and responsibilities [53,75]. Training must also allow for active engagement with the technology [57] and provide a platform for addressing users’ concerns about the safety and reliability of equipment, to enhance confidence with new ways of working [53,75]. None of the studies mentioned the use of simulation as a training approach.

## Discussion

A review of the literature was conducted to identify risks to patient safety associated with telecare use in homecare services and to investigate whether and how these patient safety risks have been addressed in telecare training. The review found a dearth of telecare studies specifically designed to study patient safety. Only one article had the investigation of patient safety as a study aim, while two other studies looked at risk management and human factors issues respectively. This shortage of telecare literature from the homecare setting with a focus on patient safety is in line with previous reviews on patient safety risks associated with the broader use of ICT-assisted healthcare devices and services [15,18]. As noted in the previous studies, risks to patient safety are frequently seen as operational challenges or as a consequence of flawed implementation. Where patient safety is mentioned as a concern, risks are often not explicitly identified or expanded upon. Though there is an emergent discussion on patient safety concerns related to the use of telecare services in the home [78], there is a distinct lack of a patient safety discourse in much of the scientific literature on telecare. Furthermore, the literature clearly lacks descriptions of how patient safety issues are being addressed in telecare training. Both findings are problematic and may hinder knowledge and understanding of how to enable provision of safe telecare services and the development of future best practices [2].

Despite not being termed as such, risks to patient safety associated with telecare do emerge in the literature, where problems and challenges associated with the implementation and use of telecare are frequently described. A reframing of these problems and challenges with the aid of a human factors systems approach to quality and safety in healthcare [12,28] helps to categorise and explicate these issues as patient safety concerns. Reconceptualising noted clinical practice issues by reference to an established patient safety framework in this way has been advocated as a method to enhance understanding of patient safety challenges and build safer care processes [79]. In light of the SEIPS model developed by Carayon and colleagues [12], patient safety risks emerging from the telecare literature are most prominently concerned with factors related to persons, tasks and technology and tools. Concerns to do with organisational factors and environmental context are also noted, though they appear less prominent. The following discussion will expand upon these findings, while also taking note of the patient safety issues that are not mentioned in the telecare literature.

### Persons

Consideration of the personal characteristics and capabilities of patients, informal caregivers and homecare staff involved in healthcare processes, including their cognitive, perceptual and physical abilities, needs, and limitations is crucial in avoiding threats to patient safety in the homecare setting [22,80]. A lack of knowledge, skills and understanding necessary to use telecare devices as intended is a pervasive safety issue identified in our review. Not being able to use telecare tools properly to successfully engage in necessary self-care or communication with healthcare providers can have potentially serious consequences for patients’ health and well-being. Safety issues contingent on a patient’s affect, such as the anxiety and dependency identified in this study have also been noted as a frequent challenge to the provision of safe home-based healthcare [80]. Furthermore, a lack of understanding of the functionality and performance of health ICTs can compromise staff and patients’ motivation and willingness to engage with telecare tools [22], resulting in compliance and adherence problems which can have a negative impact on patient safety as well [15,18].

### Tasks

A sound grasp of the nature of provider tasks and care practices is vital for examining and understanding patient safety risk in the homecare setting [22]. The patient safety issue most frequently identified in our review was where the use of telecare changed the nature of clinical work, mainly due to the physical distance created between provider and patient. In-person visits in the patient’s home and ‘knowing the patient’ through physical presence, touch, visual observation and verbal communication [30,81] is commonly regarded as fundamental for safe healthcare practice. Telecare use may however limit observational abilities and change the way providers perceive and interact with patients [30], necessitating new ways of working for healthcare staff [36,82] that are not always compatible with established means of providing care and which can be experienced as a threat to conventional clinical roles and expertise [83]. It has been argued that concerns over the safety and efficacy of telecare is centred on this loss of conventional means of knowing and caring for patients [30], where potential risks to patient safety emerge as a result of providers being unable to detect changes in patients’ health status, or making inappropriate clinical decisions [84]. Utilisation of telecare technologies necessitates reconsidered understandings of safe and appropriate care, including adaptation of practice to facilitate and support ‘knowing the patient’ via ICT-assisted healthcare processes [30].

Another task related patient safety issue that featured prominently in the literature was changes to staff workload. Alterations to traditional workflows and workloads are a common consequence of the introduction of new ICT tools and devices to the home healthcare setting [22]. Task-level workload in particular is seen as an increasingly key factor in the quality and safety of healthcare [85], as workload issues can affect patient safety in a number of ways. For example, as was seen in some of the review studies, a heavy workload affects both the total time available for tasks and the capacity to perform those tasks in a safe and timely manner. A heavy workload can also create unsafe patient care conditions by contributing to a higher likelihood of performance lapses or mistakes and errors in decision-making [86]. Several of the included studies noted that new technology necessitated a need for faster response times and more rapid decision-making. Lastly, it is important to note that workload pressures are necessarily related to patient safety at a systemic, organisational level too, as workload issues experienced by one care provider can affect others throughout the organisation [86].

### Technology and tools

Technological problems and inadequate device quality can adversely influence patient safety by resulting in ineffective use of devices and services [22]. Poor usability and/or reliability of ICT systems and devices was a noted problem in several of the included studies, as was poor technical quality of the devices used. Such problems may lead to the discontinuation or abandonment of telecare services, as was observed. The review process also revealed that while problems with telecare technologies are often described in the research literature, there is frequently no mention of the effects of these problems on patient care, safety or the clinical usefulness of telecare. It is therefore difficult to know the full extent of the impact of technology issues on patient safety risk. What appears to be a lack of adverse effects on care may in some cases be more complex, as seen in the study by Young et al., where patients actively minimized problems they encountered with the technology and assumed blame when things were not working properly [76]. More research is needed to clarify the extent to which the telecare technology itself is a threat to patient safety and the circumstances under which the technology can become unsafe.

### Organisation

The specific organisational structures and conditions under which care is organised, managed and delivered have a critical bearing on the safety and efficacy of that care [28]. One organisational factor of note in this review was to do with the scheduling and coordination of access to both telecare services and device support systems. Timely access and adequate systems for user support is crucial to the provision of safe and reliable healthcare services. Another example was poor system integration and a lack of interoperability between new telecare systems and existing ICT systems, which can limit provider access to data needed for safe clinical decision-making [2]. Perhaps the most concerning finding regarding organisational factors is the pervasive lack across provider organisations of recognised standards and procedures for service delivery that is evident in the telecare literature [77]. Provision of operational guidelines and protocols is of primary importance in ensuring safe implementation and delivery of all ICT-based healthcare services [26]. The need to apply professional practice standards and have formal procedures and protocols govern home-based telecare services is evident, so as not to seriously compromise the safety and quality of patient care. Overall, organisational factors were not prominent in these review findings, perhaps reflecting a gap in the literature. Future research on organisational aspects of safe telecare practice could focus on the role played by organisational culture and climate, management and support structures, and organisational readiness for change [87].

### Environment

Consideration of the environment in which persons, tasks and technology interact is vital to understanding these interrelations and to the attainment of safe care processes within this environment. The home environment is a complex healthcare site where both physical and social environmental factors can impact on the ability of healthcare staff to provide care [80]. In this review, only one study noted that the physical environment posed a risk to patient safety, due to the unsafe arrangement of devices in the home. As in the case of the organisational factors described above, however, the lack of findings here may not reflect a genuine absence of environmental problems related to telecare use within home-based healthcare. Rather, it is likely to reflect a gap in the literature and more research is therefore needed to ascertain the extent of environmental influences on the safe use of telecare devices in private homes, particularly the influence of socio-environmental factors such as the involvement and support, or not, of family members [80].

### Training to enhance knowledge and preparedness for telecare use

Education and training, awareness and preparedness are fundamental in ensuring safe and effective use of telecare devices. As was clear from our review however, there is an extensive lack of stakeholder knowledge and understanding to facilitate safe and appropriate telecare use. The fact that there were no studies found describing telecare training which addresses patient safety issues further underscores the importance of the development and implementation of comprehensive training and education initiatives to foster the skills, confidence and motivation needed to enable safe use of telecare services. Telecare training is an emerging field [41] and knowledge on best practice regarding the nature of required training and how to deliver it remains scarce [53]. However, the prominence in the review findings of patient safety risks respectively related to the changing nature of homecare tasks and the characteristics and capabilities of stakeholders reflect a dire need for competency building to ensure safe and effective patient care in the homecare setting. Therefore, in addition to technical skills training and ensuring that all stakeholders have a common understanding of respective tasks and responsibilities [15], it is of vital importance to focus training on development of the knowledge, skills, attitudes and experiences required for new ways of working [34,36] and to foster new thinking and practice related to ‘knowing the patient’ [30]. This will go a long way toward ensuring safe and appropriate implementation and use of telecare in the delivery of healthcare services in the homecare context.

### Limitations

There are potential limitations in our study. First, regarding the search terms and search structure used in the electronic database searches, we could have missed identifying relevant articles describing patient safety risks associated with telecare use due to the inclusion of ‘training’ and related terms in the chosen combination of search terms. However, since the combined aim of the review was to identify patient safety risks and explore how these risks have been addressed in training, it was decided to do a combined search. The dearth of studies found in this review with a focus on patient safety risk connected to the use of telecare in home healthcare services mirror results seen in similar reviews on the quality and safety of ICT use in health and social care services [15,18]. One reason for this could be the observed lack of a patient safety discourse in the telecare literature, meaning that relevant publications could be missed by traditional database searches due to not being indexed in such a way as to allow identification within the parameters of such a search [88]. This is a major reason for also conducting hand searches and searches based on reference tracking and citation tracking, to increase the chances of finding further relevant items [52].

Secondly, the exclusion of studies featuring children as recipients of telecare services can be considered a limitation of this study. The reason for this exclusion is the context of the review study, which is part of a larger research project focused on telecare services for the elderly and the development of training initiatives for healthcare professionals who work with adult and elderly service users [46]. While relevant patient safety risks could well have been overlooked with the exclusion of paediatric telecare studies, the focus of this review was adult patients as users of telecare rather than children and their families. It could be of interest to future research to investigate whether and how risks to patient safety differ in telecare services for the paediatric population.

Thirdly, the review findings reflect the types of studies included, the methods used and the subjective, experiential data thus generated. Most of the included studies have a descriptive, exploratory design, use qualitative methods and feature data on views and experiences of patients and healthcare staff. Some of the identified patient safety risks are thus reconceptualization of subjective, experientially-based opinions of various telecare users, and not objective measures of risk as such. This could be considered a weakness. However, these findings do reflect the aforementioned importance of considering user characteristics, needs and experiences in conceptualising and understanding patient safety risk related to telecare use, as well as the fundamental significance of the nature of provider tasks and roles and responsibilities of healthcare staff. These are concerns and perspectives that have traditionally been excluded from patient safety frameworks and there have therefore been calls for increased consideration of user, carer and provider views and experiences in conceptualisations of patient safety issues in the homecare context [22].

Finally it must be stressed that studies which reported on participants’ feelings of being safe and secure when using telecare services in the home were not included in the review. Feeling safe as part of the experience of using a telecare service is not the same as not being exposed to patient safety risks as the user of that service and the service actually delivering quality of care. Similarly, the search identified a number of articles where telecare related clinical risks and patient safety issues were noted as a major concern, particularly among healthcare professionals, but where there was no elaboration upon what these risks are and how they may affect patient care and safety. These studies were also excluded.

## Conclusion

Patient safety risks associated with telecare use are frequently not framed by a patient safety discourse. Reframing described telecare implementation challenges and user experiences by reference to a human factors systems framework of patient safety, such as the SEIPS model [12], has enabled identification and discussion of potential safety threats associated with the use of telecare in the home healthcare setting. Efforts to improve identification of safety and quality issues will hopefully lead to further enhanced understandings of the patient safety risks related to telecare, including more knowledge of direct and latent antecedents to such risks. It will also facilitate learning and competency building, alongside the development of best clinical practice for further mitigation of potential harm [18,30]. A human factors systems approach emphasises the systemic factors that underlie identified risks. While considerations of individual contexts and meanings of use clearly are important to ensure safe and successful use of ICTs in healthcare, patient safety issues at all levels are embedded within overarching cultural, social and political structures and circumstances that govern healthcare in the complex home environment [22]. It is important to recognise, therefore, that the mitigation of patient safety risks, whether to do with the people, tasks or the technology involved, are likely also dependent on these broad-ranging systemic parameters of telecare services [2,89].

Sound stakeholder knowledge and understanding of telecare systems and related services emerges as a major prerequisite for their safe use. Telecare training for all telecare users including healthcare professionals should address a wide variety of concerns to increase awareness of potential patient safety risks and should furthermore prepare healthcare staff for new ways of working. Training and education that raises awareness of safety and quality issues can thus promote user confidence and skill in the provision of safe telecare services, thereby aiding the minimization of potential harm to patients associated with the introduction of telecare services. Aside from appropriate training initiatives, there is also an urgent need for system-wide professional protocols, clinical practice guidelines and quality assurance systems to guide and assess the use of telecare in the complex domestic setting.
